# 

**Published:** 1842-05-07

**Authors:** 


**CONTENTS OF No. 19.**

Dr. S. Lewis' Case in which Alcohol was detected in the Brain, . . 289
Directions for Analysing the Brain to discover Alcohol, . 290
Presence of Alcohol in the system after poisoning from Alcoholic Liquors, . . . . . . . . . 292
Dr. George F. Carmichael on the Treatment of Obstinate Constipation, with cases, . . . . . . . . . . 294
ANALECTA: Mr. Procter's Observations on Hydrated Peroxide of Iron, 295
Report on the Nutritive properties of Gelatine, . . . 299
M. D'Arcet on the employment of Gelatine and Fat of Bones as a means of ameliorating the diet of the poor, . . 300
The Commission of the French Academy on the Ferruginous Bread of M. Derouet-Boissière, . . . . . 300
Mr. Mill's Case of Diarrhœa from Ulceration of the Intestinal Glands cured by the internal use of the Chloride of Sodium, 301
Mr. Graham's Case of Cramp in the Stomach, . . . 302
Dr. Sutherland on Undiluted Creasote in Burns, . . . 302
M. Pigeau's Case of Asphyxia dissipated by a pinch of snuff, 303
M. Huguier on Croup in the Adult, . . . . . 303
Dr. Ducros' Experimental Researches on the Function of the Skin in man and animals, . . . . . . . 303
M. Begin on the Treatment of Hæmorrhage after Lithotomy, 304

